# Dynamic Hydrogel‐Based Strategy for Traumatic Brain Injury Modeling and Therapy

**DOI:** 10.1111/cns.70148

**Published:** 2025-01-09

**Authors:** Xin He, Meng Lei, Xuewen Chen, Feng Xu, Heng Liu, Zhao Wei

**Affiliations:** ^1^ Department of Radiology Affiliated Hospital of Zunyi Medical University, Engineering Research Center of Intelligent Medical Imaging in Guizhou Higher Education Zunyi People's Republic of China; ^2^ The Key Laboratory of Biomedical Information Engineering of Ministry of Education, School of Life Science and Technology Xi'an Jiaotong University Xi'an China; ^3^ Bioinspired Engineering and Biomechanics Center (BEBC) Xi'an Jiaotong University Xi'an People's Republic of China

**Keywords:** dynamic hydrogels, dynamic network, stiffness changing, traumatic brain injury

## Abstract

Traumatic brain injury (TBI) is one of the most traumatizing and poses serious health risks to people's bodies due to its unique pathophysiological characteristics. The investigations on the pathological mechanism and valid interventions of TBI have attracted widespread attention worldwide. With bio‐mimic mechanic cues, the dynamic hydrogels with dynamic stiffness changes or reversible crosslinking have been suggested to construct the in vitro disease models or novel therapeutic agents for TBI. However, there is a lack of clarification on the dynamic hydrogels currently reported and their biomedical applications on TBI. Our review starts with introducing the native mechanical characters and changes in TBI and then summarizes the common chemical strategies of the dynamic hydrogels with dynamically tunable stiffness and reversible networks for in vitro modeling and therapy. Finally, we prospect the future development of dynamic hydrogels in the mechanical modeling of TBI, providing new mechanical insights for TBI and guidance for tailored brain‐targeted biomaterials.

## Graphical abstract

Dynamic hydrogels are often defined as dynamic network hydrogels and dynamic stiffness hydrogels, and noncovalent versus dynamic covalent chemistry is usually applied as a common preparation strategy. We can not only apply dynamic hydrogel‐loaded neural cells to construct an in vitro model to simulate the process of traumatic brain injury (TBI) but also implant dynamic hydrogel as a scaffold or medium loaded with neural cells into the core of TBI injury to treat TBI and promote nerve repair.

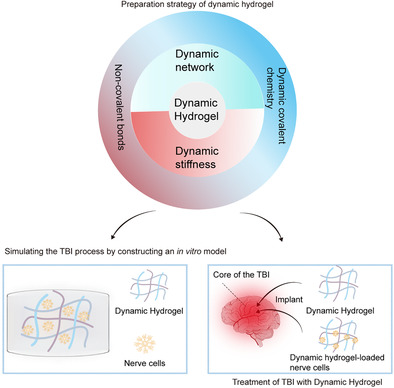

## 1 Introduction

Traumatic brain injury (TBI) is one of the most prevalent types of brain injury and causes persistent morbidity and substantial mortality across all countries and age groups [1]. The deterioration of TBI will induce massive death of functional neuronal cells in the focal area and dramatic loss of extracellular matrix (ECM) forming a cystic cavity [2]. Currently, the clinical treatment options for TBI are oral medication and hematoma evacuation, but both are indirect interventions, and their therapeutic effects on severe injury are still very poor [3]. Therefore, there is an urgent need for an effective treatment strategy for TBI.

Glial scarring plays a role in maintaining homeostasis in the brain in the early stages after brain injury [4]; however, studies have revealed that the inability to regenerate brain tissue following TBI leads to the formation of a cavity. The TBI results in immediate tissue loss and cavity formation, with previous approaches to simulate the pathological features of TBI mainly based on live animal models, involving ethical and technical issues such as training and monitoring of animal models [5, 6]. Occurring subsequently, typically initiating approximately 7 days after injury [7, 8, 9], it inhibits axon regeneration in the later stages and hinders nerve repair [9]. As a result, effective methods for inhibiting cavity enlargement and minimizing glial scar formation would provide a broad therapeutic prospect for nerve repair [10, 11]. However, after TBI, the biochemical and physical microenvironment of the lesion is altered, especially the local ECM stiffness change caused by the glial scar formation around the core area of the lesion, resulting in low therapeutic cell efficiency and a physical barrier to nerve tissue regeneration [12, 13]. Moreover, the filling of engineered materials with no mechanical similarity in brain organization is detrimental to therapeutic cell functions and the recruitment of local neuronal cells for nerve regeneration. As a result, numerous efforts have been proposed to investigate the effects of ECM mechanics on TBI by constructing both in vitro models and in vivo fillers [14, 15].

Moreover, previous studies have applied hydrogels to model TBI in vitro to simulate postinjury changes [16, 17, 18], for example, porous, topologically complex silk protein scaffolds constructed in vitro to study the pathophysiology of cortical TBI. Therefore, the preparation of hydrogels is used to construct in vitro models similar to TBI [17]. So, the preparation of hydrogels is used to construct in vitro models similar to TBI, which were used to simulate the process of TBI and understand the pathophysiological changes. However, TBI models based on dynamic hydrogels are rarely reported. Therefore, one of the purposes of this review is to summarize and discuss the dynamic hydrogels currently applied to TBI models.

Recently, the design and development of dynamic hydrogels have attracted increased attention in TBI modeling and treatment. The implication of “dynamic” has been divided into two explanations. One is the hydrogel with dynamically tunable stiffness (denoted as dynamic stiffness hydrogels, DSH) which aims to establish an in vitro model for the study of the effects on cells of the process of pathological stiffness changes in TBI. Another one is the hydrogel crosslinked by dynamic networks (denoted as dynamic network hydrogels, DNH), which aims to imitate the viscoelasticity of the brain and improve the efficiency of TBI treatment. Although plenty of the above studies have been reported and both have been called dynamic hydrogels, there is a lack of clarification on their design strategies and applications, as well as their joint effects on TBI modeling and treatments, remain largely unknown, which poses an urgent need for a review of these.

In this review, we first introduce the mechanical cues of the normal brain and the changes in stiffness after TBI. We then summarize the design and development of dynamic hydrogels based on TBI modeling and therapeutic strategies. Finally, we provide a perspective on the use of dynamic hydrogels in TBI treatment. This review will not only help us deeply understand the underlying mechanism by which cells respond to mechanical cues during TBI development and recovery but will also provide a more refined design of tailored dynamic hydrogel systems and broaden their applications in brain tissue engineering.

## 2 The Mechanical Microenvironment of Brain Tissue During TBI

**FIGURE 1:**
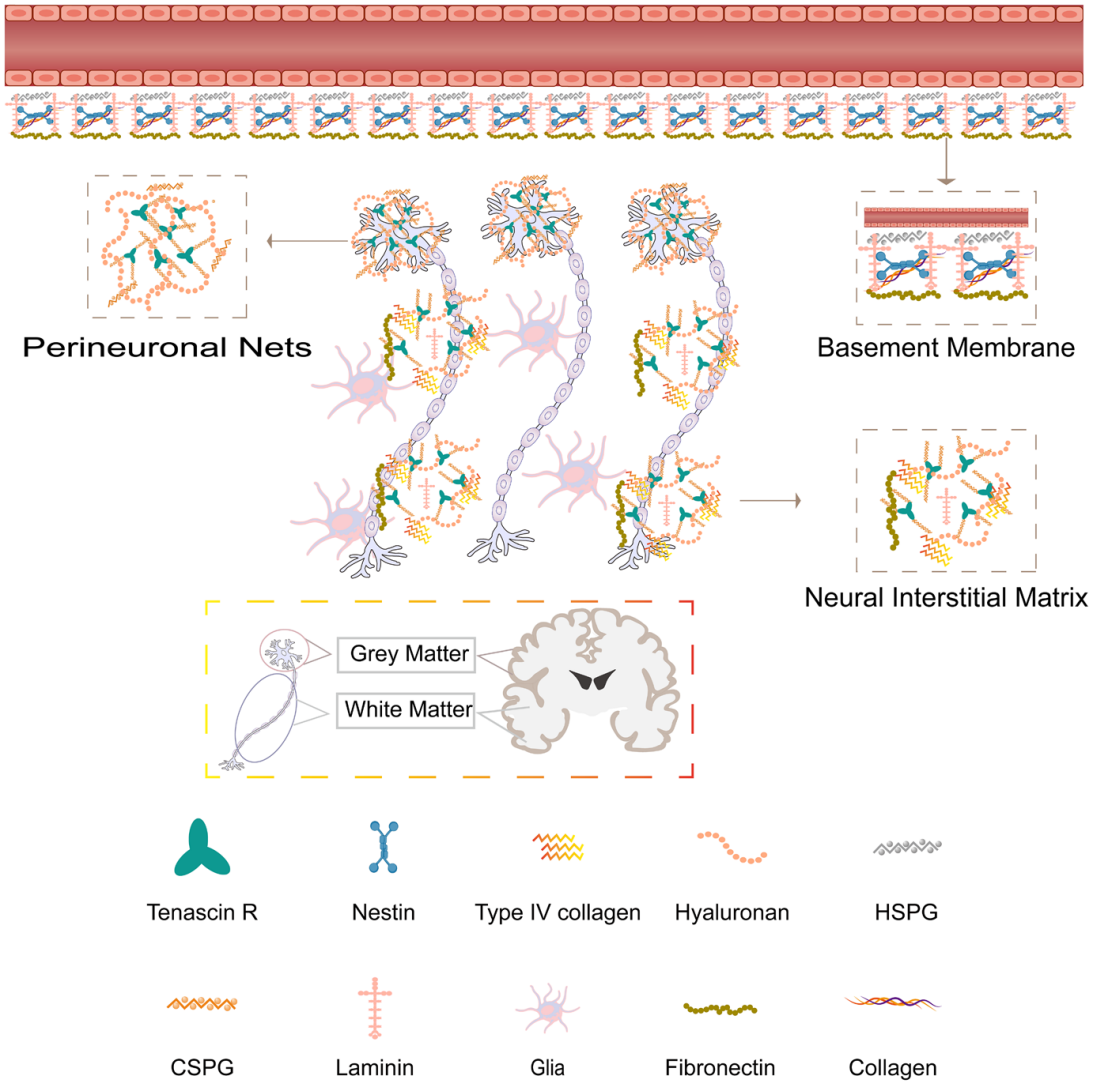
The major compartments of the ECM in the CNS, and the spatial distribution of white matter and gray matter. ECM components include basement membranes that lie outside cerebral vessels, the peripheral neural network condenses around the cell body, and the nerve interstitial matrix which is distributed between parenchyma cells.

Brain tissue is composed of neuronal cell bodies and their processes (dendrites and axons), and is interconnected with ECM, blood vessels, glial cells, and extracellular fluid [19]. The ECM is about 20% of the brain volume and mainly helps provide mechanical support for the brain [20], which mainly consists of collagen, proteoglycans glycoproteins, etc. [21]. As Figure 1 shows, the central nervous system (CNS) contains the basement membrane, the perineuronal networks, and the interneurons [22]. The basement membrane, arranged on the parenchymal side of the cerebral microvasculature, is a continuous structure composed mainly of type IV collagen, laminin–nestrin complex, fibronectin, and heparan sulfate proteoglycans (HSPGs) [23, 24], which plays an important role in neuronal and neuroglia function and building and keeping cell differentiation and polarity [25]. The peripheral neural networks are tightly wrapped in the cell bodies and dendrites of certain neurons with tenascin‐R, (HA), chondroitin sulfate proteoglycans (CSPGs), and connectins, which promotes synaptic stability in the adult brain [26, 27]. The interneuron consists of parenchymal ECM ingredients, embodying proteoglycans, HA, sequestrants, and connexins, as well as relatively small amounts of fibronectin and viscous glycoproteins [27]. The formation of the ECM mentioned above in brain tissue gives it inimitable mechanical properties of stiffness and viscoelasticity.

The mechanical stiffness of living tissues varies greatly and exhibits a very wide range of modulus variations from brain tissue to bone tissue [28]. The stiffness of the brain brings about a direct effect in regulating neuronal network activity, and regulatory processes depend on specific types of ECM proteins. The values of modulus measured in different brain regions or by different testing approaches vary but are mostly within 10^2^–10^3^ Pa [19, 27, 29, 30, 31, 32]. In particular, the stiffness of brain ECM notably influences the differentiation of neural stem cells, and oligodendrocytes [33]. At a low modulus of elasticity of the matrix, the cell adhesion is weakened and diffusion is enhanced [34, 35], whereas a matrix with a high modulus promotes dendritic differentiation [36]. For example, softer substrates are more favorable for the growth of cortical neurons, and neurons are more likely to branch on softer substrates [37]. In contrast, astrocyte growth is promoted on stiffer substrates [38].

After TBI, the mechanical stiffness of the brain ECM is greatly altered since the reactive astrocytes secrete large amounts of CSPGs, growth factors, and inflammatory cytokines, as well as the levels of HA and fibrillar collagen (e.g., collagen type I/IV) are significantly increased. The correspondingly decreased stiffness of the brain matrix promotes glial scar formation, prevents myelin regeneration, and inhibits neurofunctional recovery [39, 40, 41]. For example, Franze et al. [38] found that cortical stiffness can range from ~10^3^ Pa (healthy tissue) to nearly ~50 Pa (scarred area) during glial scarring after brain injury, which may be related to the expressions of intermediate filaments (e.g., GFAP and Wave protein) and ECM constituents (e.g., laminin and collagen type IV). The upregulation and highly hydrated expression of proteoglycan components (e.g., CSPGs) in the ECM of glial scarring promote local tissue softening [42], and the upregulation of collagen IV and laminin associated with vascular rupture also leads to decreased tissue stiffness [43].

Besides stiffness, the brain ECM also exhibits fast relaxation of stress over time [44]. Different instruments including magnetic resonance elastography and AFM can be applied to test the viscoelasticity of the brain [27, 45]. The stress relaxation of the brain tissues has also been found to regulate the stem maintenance and maturation of neural progenitor cells (NPCs) [46]. In addition, an increased viscoelasticity has been observed in scar‐forming regions after TBI [47].

## 3 Dynamic Hydrogel Strategies for Modeling and Treating TBI

**FIGURE 2:**
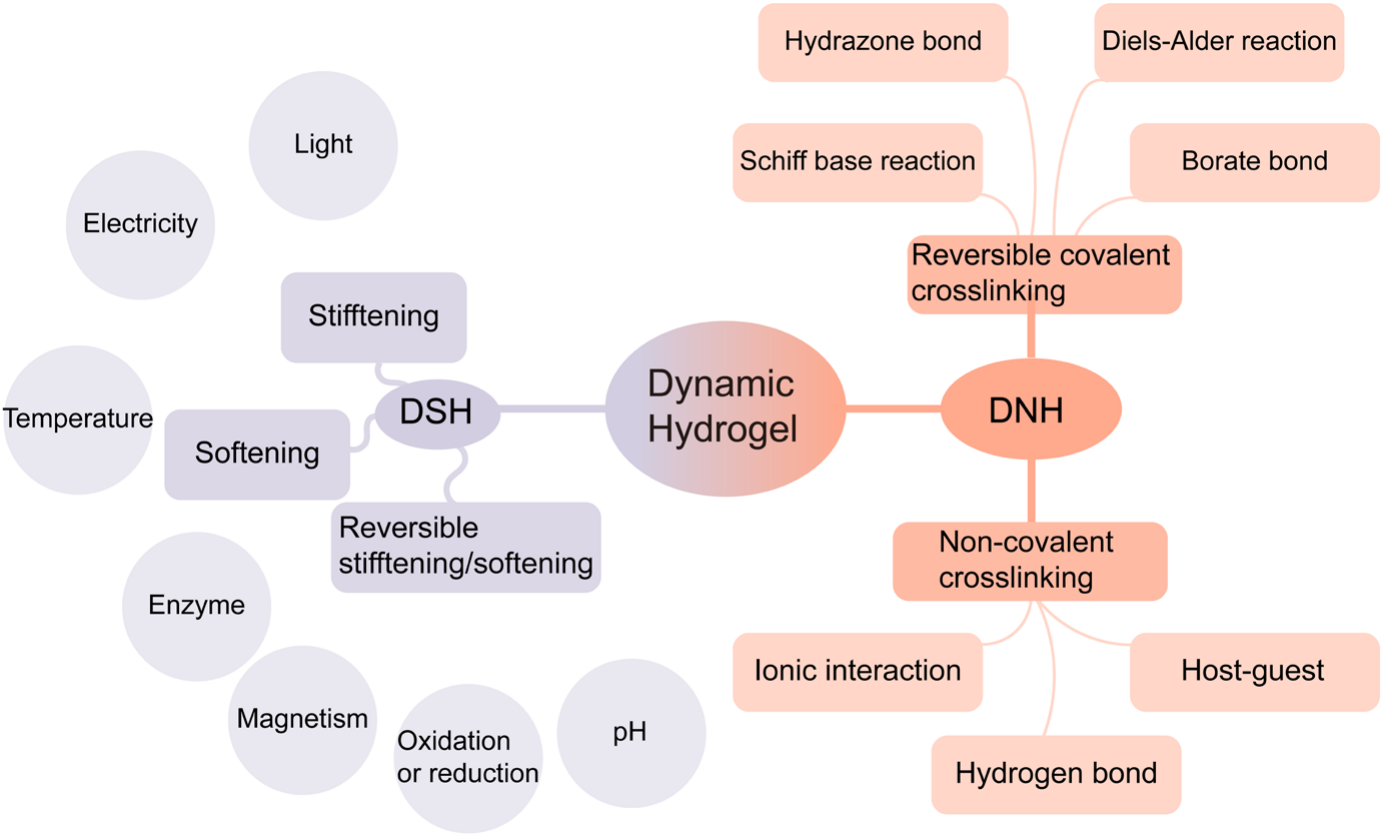
Common strategies for preparing dynamic hydrogels for DSH and DNH. The common formation of dynamic network hydrogels is mainly based on reversible covalent crosslinking and noncovalent crosslinking. The design of dynamically tunable stiffness mainly uses light, temperature, pH, enzyme, electrical or magnetic response, oxidation or reduction, etc. to harden or soften the hydrogel, or even achieve reversible dynamic softening/hardening.

Hydrogels with 3D water‐soluble network structures can mimic the microenvironment of living brain tissues, and have great capacity to be a candidate biomaterial for brain tissue engineering. We introduce the design and preparation strategies of the tailored dynamic hydrogels of DSH for TBI modeling and DNH for TBI filling treatment (Figure 2 and Table 1).

**TABLE 1:** Application of dynamic hydrogel for in vitro modeling and treatment of TBI.

Preparation materials of DSH hydrogel	Stimuli	Stiffness variation	Application of in vitro model	Refs
Alginate, collagen Type I, calcium ions	Regulation of crosslinking and dissociation of alginate with calcium ions	42.7 Pa ~ 307.6 Pa ~ 990.6 Pa	Simulating the dynamic softening process of glial scarring in TBI	[47]
DNA strand, polyacrylamide gel	Adding a toehold to the DNA strand changes the crosslink density.	—	Investigate the response of nerve cells to changes in substrate dynamic stiffness	[48]
**Preparation materials of DNH hydrogel**	**Stimuli and crosslinking method**		**Treatment of TBI with DNH hydrogel**	
Polyethylene glycol‐poly (n‐isopropylacrylamide) hydrogel	Temperature, physical crosslinking	—	Treating demyelinating diseases and brain injuries.	[14]
Electrospun fiber, agarose/methylcellulose hydrogel	Temperature, physical crosslinking	—	Promote the regeneration of injured brain tissue	[49]
ECM‐hydrogel	Temperature, physical crosslinking (the material of ECM is mostly collagen)	—	Implant the injured site to promote the repair of white matter and improve nerve function.	[50, 51, 52]
Hyaluronic acid‐chitosan hydrogel	Temperature, Schiff base reaction	Stiffness increases with temperature at 25°C–37°C	Provide a microenvironment for axonal growth	94
Based on hyaluronic acid, add nerve cells or BDNF	Covalent crosslinking, physical crosslinking, dynamic covalent crosslinking	100 Pa, 350 Pa, 1000 Pa	Inject to the injured area to improve nerves	[53, 54, 55]
IKVAV+ multifunctional peptide hydrogel + neural stem cells	Noncovalent bond	3Kpa–10Kpa	Reduce glial scars and enhance the reconstruction of damaged brain tissue	[56]
Gelatin‐modified chitosan/β‐glycerophosphate hydrogel	Temperature, physical crosslinking	—	Delivery of medication for TBI treatment	[57]
Alginate, sterculia gum polysaccharide hydrogel	Hydrogen bond	—	Brain drug delivery	[58]

### 3.1 The Design of DSH and Their Applications in TBI Modeling

The pathologic changes of TBI are extremely complex, which limits our research on disease onset, progression, and treatment. Therefore, it is a breakthrough to apply hydrogels to build in vitro models that are as close as possible to brain tissue. Former studies have found that the stiffness of brain ECM is a dynamic softening process during glial scarring after TBI [38]. Therefore, it is necessary to design DSH with dynamic stiffness changes to simulate dynamic modulus changes in disease to more accurately explore the interactions between cell and matrix changes.

To dynamically vary the stiffness of hydrogels, it is common to alter the crosslinking density or the dexterity of chain segments [59]. The DSH with increasing stiffness is often designed to allow for secondary crosslinking when exposed to outside irritants (e.g., light, temperature, pH, or enzymes) [60]. There are several exhaustive studies below for elaboration: Nicholas et al. [61] synthesized hydrogels based on DNA strands and GelMA, which were softened by adding replacement strands with fully complementary crosslinks to reduce the crosslinking density and then adding new crosslinking agents to increase the crosslinking density reversibly increased the stiffness of the hydrogels. Similarly, Wu et al. [62] prepared a double‐network hydrogel of polyvinyl alcohol (PVA)/alginate with hydrogen bonding and ionic bonding, where PVA and alginate were connected by hydrogen bonding, and the subsequent addition of calcium ions, which ionically interacted with the alginate, increased the concentration of calcium ions, and the ionic crosslinking density increased, which ultimately led to an increase in the stiffness and the ionic crosslinking density was decreased if the calcium ions were chelated and the stiffness was reduced. Application of thiolated PEG and nitrobenzene to prepare hydrogels via thiol–epoxy coupling reaction, followed by phototriggering principle to reduce crosslinking density dynamics by UV irradiation for dynamic softening of the hydrogels [63]. Dynamic hardening of hydrogels by incubating initial hydrogels in aldehyde‐containing macromolecules and changing the crosslinking density of hydrogels through the simultaneous application of the mercapto‐norbornene photoclick and hydrazone click reactions [64].

In recent years, several applications of light‐responsive hydrogels have been reported [65, 66, 67, 68, 69]. Light‐responsive host–guest hydrogels have also been widely used, in which light‐responsive semireversible hydrogels are prepared based on the host–guest interactions between cyclodextrins (CDs) and azobenzenes (AZOs) to achieve dynamic hardening/softening [70, 71]. Moreover, Kim's team developed chitosan/b‐glycerophosphate (Ch/b‐GP) thermosensitive gels with a combination of flexible and semiflexible networks, with the flexible network collapsing upon heating for transient hardening [72]. Liang et al. [73] skillfully applied the electrical modulation method to control the hardness gradient of alginate gels to soften or harden based on the principle of ionic action. Hydrolysis and enzymatic hydrolysis provide softened hydrogel materials that are highly tunable and widely adaptable. Shi et al. [74] prepared enzyme‐degraded PEG hydrogels with adjustable degradation rates, whose mechanical properties decreased with increasing degradation crosslinker content. Jones' team developed hydrolyzable degradable composite hydrogels based on polyethylene glycol and HA [75]. Murat applied HA to prepare DSH, which was hardened in situ by a continuous crosslinking reaction of methacrylic anhydride initiated by light [76]. The above DSH systems not only deepen the understanding of the mechanism of hydrogel hardness regulation but also provide a powerful material tool for studying TBI.

**FIGURE 3:**
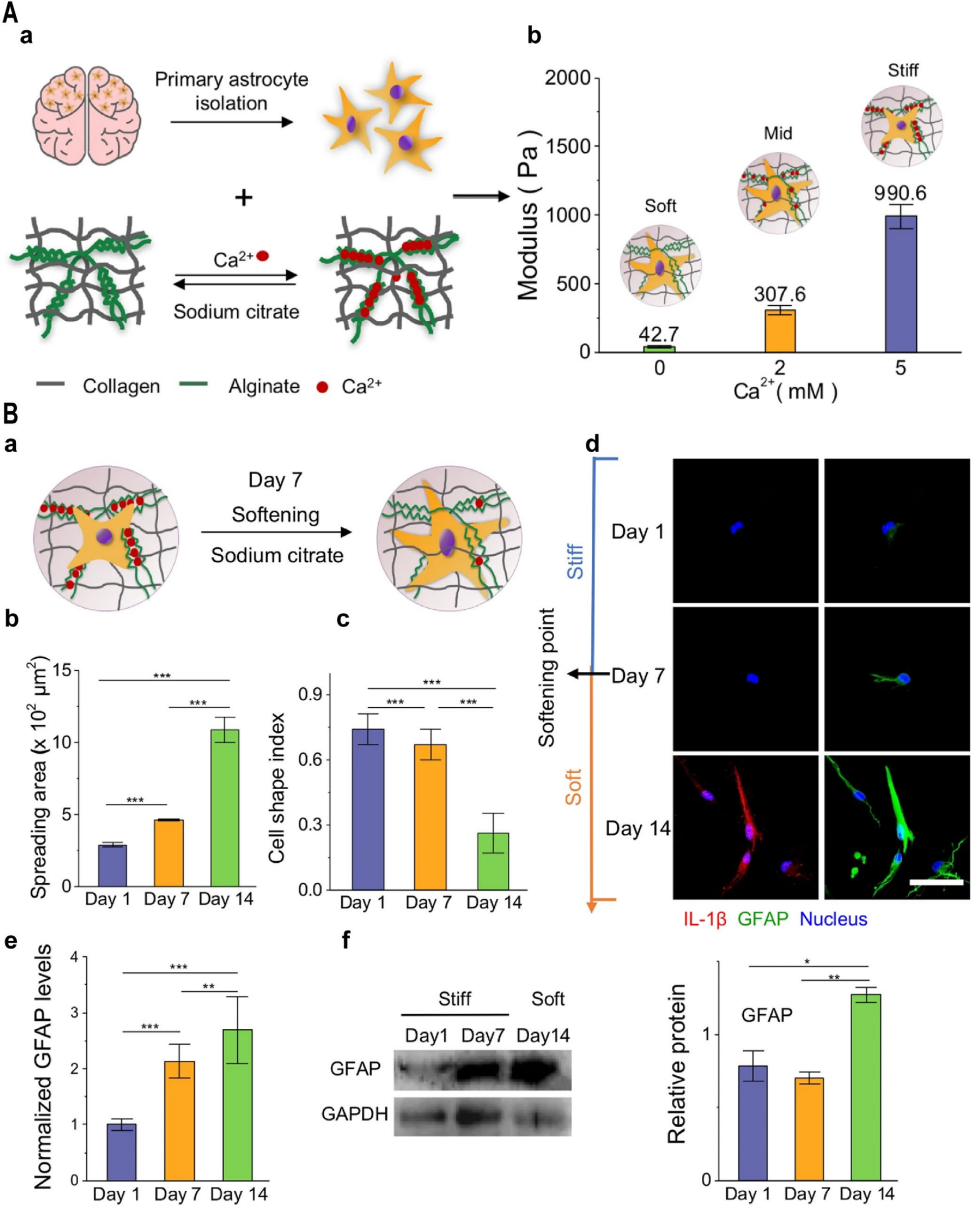
Dynamic stiffness tunable hydrogel constructs a TBI model with a glial scar softening process that induces astrocyte activation. (A) Schematic preparation and mechanical property characterization of in situ dynamically adjustable stiffness hydrogels with stiffness‐matched brain tissue mechanical properties. (B) Effect of stiffness softening on astrocyte phenotype during glial scarring. (b, c) Increased spreading area of astrocytes and morphological changes due to matrix softening (reactive glial cell hypertrophic morphology). (d–f) Matrix softening significantly upregulates GFAP and IL‐1β expression. Scale bar: 50 μm. *P < 0.05, **P < 0.01, ***P < 0.001, and ****P < 0.0001. Adapted and reprinted with permission from Ref [47].

The widespread use of DSH to simulate the stiffness changes after TBI helps us understand the interactions between neural cells and their mechanical environments. Jiang's team developed a DSH system in which they attached DNA to strands of a polyacrylamide gel by covalent crosslinking, and when a stub or complement was added to the DNA strand, gelation or de‐gelation occurred for dynamically modulating stiffness, and the hydrogel was accepted as the method to research the responsiveness of the nerve cellular response to changes in stiffness in the dynamic stiffness of the matrix [48]. Our group applied alginate to crosslink with calcium ions, and subsequently used sodium citrate to chelate calcium ions to achieve in situ dynamic softening of the DSH hydrogel system to simulate the dynamic softening process of glial scarring in TBI, and we revealed the effect and mechanism of substrate stiffness softening on astrocyte phenotypic changes in brain injury ([47]; Figure 3). In addition, a complex hydrogel made up of a network of rigid polysaccharide networks (pectin and sodium alginate) and a flexographic polyacrylamide network can well mimic the altered mechanical properties of the hindbrain of TBI through chemical and physical bond crosslinking [77].

### 3.2 The Design of DNH and Their Applications in TBI Treatments

The brain is one of the softest and most dissipative viscoelastic tissues in mammals [78]; this study has demonstrated that the native brain tissues are viscoelastic with a stress relax rate in the range of ~1 s. DNH can be used as bio‐mimic carriers and scaffolds for transporting substances to treat TBI. The implanted DNH not only reduces lesion size and improves neurobehavioral functions [50]. The viscoelasticity of DNH can be influenced by the different kinetic constants of the dynamic reactions. Studies have shown that the equilibrium binding constant (Keq) and kinetic binding constants (koff/k‐1 and kon/k1) play crucial roles in determining the stiffness and viscoelastic properties of hydrogels that incorporate reversible or dynamic crosslinks. A higher Keq indicates stronger or more stable crosslinks because the binding affinity between the network's interacting molecules is higher. Higher koff means that the crosslinks are more dynamic, dissociating rapidly under stress, which allows the hydrogel to relax stress more quickly, leading to more pronounced viscoelastic behavior [79].

Cells embedded in static hydrogel networks crosslinked by covalent bonds severely impede cell–substrate interactions and normal cellular functions. There have been numerous studies demonstrating that more viscoelastic hydrogels lead to greater focal adhesion and longer lifespan, contributing to neurite extension, migration, and maturation of neurons [80, 81, 82]. The DNH hydrogels consist of dynamic bonds with breaking and re‐forming reversibly, allowing them to better adapt cell functions and activities. For example, compared with irreversibly covalently crosslinked GelMA hydrogels, neural stem cells in dynamically networked hydrogels not only showed higher activity and differentiation but also the better spreading of neural cells and axonal extension within the hydrogels [83]. Therefore, using DNH hydrogels to deliver nerve cells to the site of injury has the potential to improve the therapeutic effects after TBI. Besides, the application of DNH can also minimize the harmful effects of inflammation in the early stage and promote nerve fiber regeneration [83, 84].

Currently, noncovalent or dynamic covalent chemistry has been introduced in the design of DNH as a means of dynamic crosslinking to achieve the bio‐mimic viscoelasticity of the brain tissue. Ionic interactions, host–guest interactions, and hydrogen bonds as noncovalent bonds, which are often in a dynamic state of breaking and reorganization, or in an equilibrium state of breaking when appropriate stimuli are applied. Hydrogels prepared using noncovalent bonds have reversible/dynamic sol–gel transition properties and stimulus responsiveness [85]. (1) Ionic interactions: They are usually formed by electrostatic attraction or repulsion forces between charged groups, which are characterized by relatively weak forces and dependence on environmental conditions (e.g., pH and ion concentration). As conditions change, ionic interactions rapidly dissociate and reorganize, and this reversible dynamic change characteristic allows the hydrogel to adjust its internal structure when subjected to external stimuli, permitting the material to maintain its overall integrity while possessing properties such as self‐healing and deformation adaptation. Thus, ionic interactions provide flexibility and tunable mechanical response to hydrogels. (2) Host–guest interactions: It provides dynamics in dynamically crosslinked hydrogels through its characteristic reversibility and selectivity. Reversible encapsulation is formed by noncovalent bonding between the host molecule (usually a ring structure, such as CD) and the guest molecule (usually a small molecule or part of a polymer chain). Because this binding is dynamic, the host–guest complex can dissociate and rebind in response to external stimuli (e.g., concentration changes, pH, temperature, or the competitive effects of specific molecules), and this controlled process of binding and dissociation endows the hydrogel with flexible and dynamic properties. (3) Hydrogen bond: The key to the dynamism provided by hydrogen bonds in dynamically crosslinked hydrogels is their reversibility. Similarly, hydrogen bonds are weak and dependent on the external environment and can be broken and re‐formed as the environment changes. This process of fracture and reorganization allows the molecular network of the hydrogel to be continuously adapted and reconfigured, endowing the hydrogel with dynamic mechanical properties such as self‐repair, deformation response, and mechanical adaptability. This dynamic behavior allows hydrogels to maintain their structural integrity and functionality when subjected to external forces or environmental changes.

The hydrogels prepared with noncovalent bonds are characterized by a reversible/dynamic sol–gel transition. The dynamic sol–gel process of hydrogels involves a change in viscoelasticity. Noncovalent bonding affects the viscoelasticity of hydrogels mainly by (1) enhancing reversibility and dynamic behavior noncovalent bonds can be formed and dissociated repeatedly, allowing the network of the hydrogel to partially deconstruct and spontaneously recover when subjected to mechanical stress. This dynamic behavior increases the elasticity and flexibility of the hydrogel while reducing the long‐term deformation (i.e., creep) of the material. (2) Improve self‐healing ability: The reversibility of the noncovalent bonds makes the hydrogel self‐healing. When the hydrogel is mechanically damaged, these bonds can be reformed in a short period, restoring its structure and mechanical properties and enhancing the viscoelastic behavior of the material. (3) Regulating the balance of viscosity and elasticity: The strength of noncovalent bonds and the rate of binding/dissociation determine the viscous and elastic behavior of hydrogels. Weaker or rapidly reorganizing noncovalent bonds (e.g., hydrogen bonds) tend to impart higher viscosity to the material, while stronger or slower dissociating noncovalent bonds (e.g., hydrophobic interactions) enhance the elasticity of the material. (4) Responding to environmental changes: The formation and dissociation of noncovalent bonds are highly sensitive to the external environment such as temperature, pH, and ion concentration. By regulating these external conditions, the viscoelasticity of hydrogels can be dynamically adjusted, which provides more controllability for their applications in fields such as biomedicine.

For example, host–guest DNH hydrogels have been widely used with tunable viscoelasticity recently [70, 71, 86, 87, 88, 89, 90, 91, 92, 93]. Their viscoelastic properties such as stress relaxation depend on the concentration of the macromolecule, the degree of modification of the guest macromolecule, and the molar ratio of the host and guest functional groups. A compound hydrogel combines brain‐decellularized ECM (dECM) and matrix metallopeptidase (MMP)‐respond‐able host–guest hydrogel being a delivery podium to enhance survival of cells and differentiation of transplanted neural stem cells [94]. However, the disadvantage of the host–guest DNH system is the instability of their crosslinks and thus the extremely low mechanical stiffness.

**FIGURE 4:**
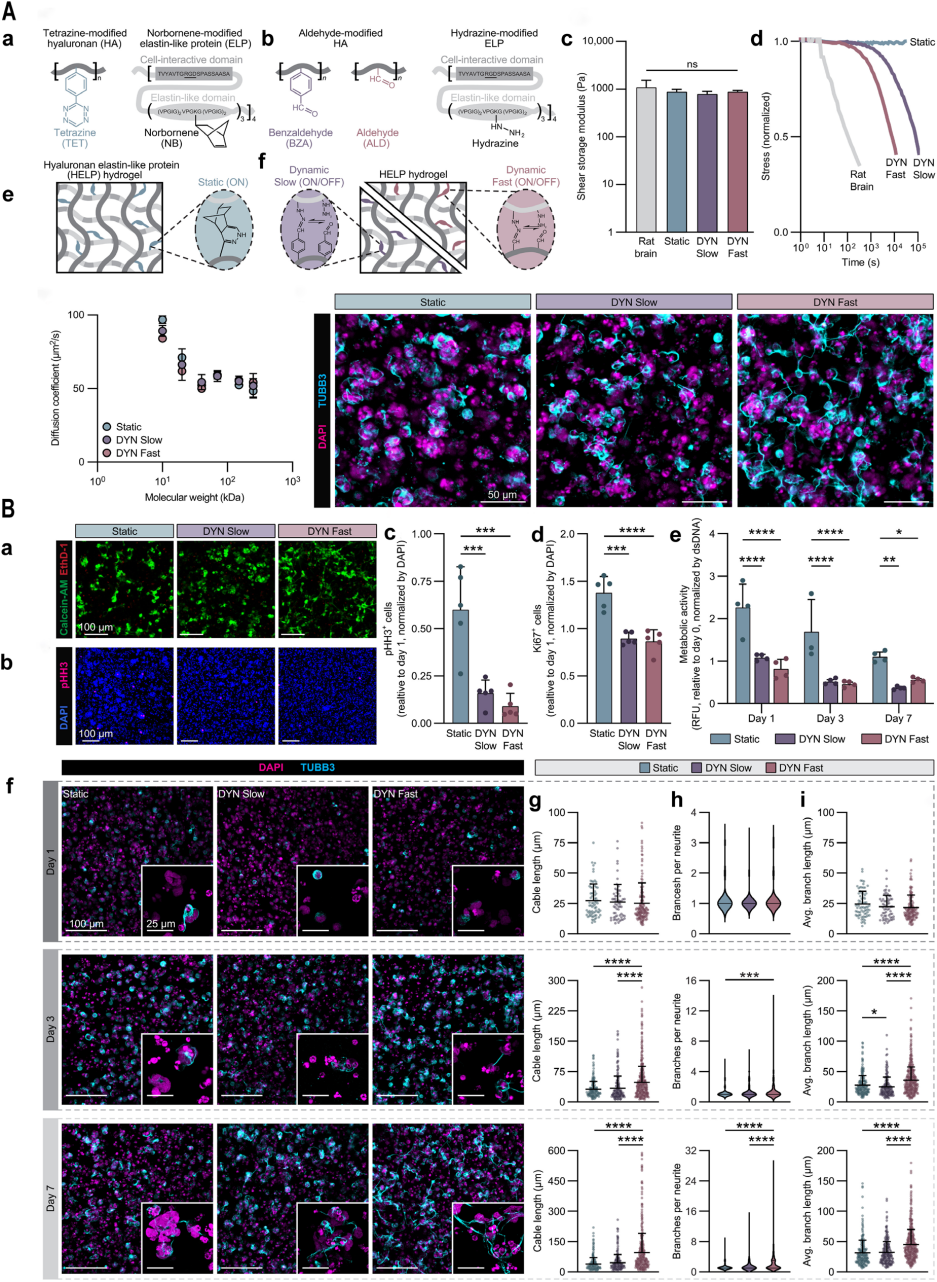
**Stress‐relaxing hydrogels promote NPC maturation**. (A) Tunable viscoelastic hydrogel promotes NPC culture and neurite extension. (a, b) Schematic representation of the preparation of static covalent and dynamic covalent crosslinked hydrogels. (c, d) Shear modulus and stress relaxation curves for rat brain and hydrogels. (e) Molecular diffusivity of hydrogels. (f) Fluorescence image of NPC extension. Scale bar: 50 μm. (B) Stress relaxation promotes greater maturation of NPC physiology and morphology. (a) NPC dead‐viable staining. (b) Fluorescence images of the proliferation marker pHH3 after 7 days of NPC culture. (c, d) Quantification of the proliferation markers pHH3 and Ki67 after 7 days of culture. (e) Quantification of the relative metabolic activity of cells over time. (f) Fluorescence images of the neuronal lineage marker βIII‐tubulin (TUBB3) on days 1, 3, and 7 of cell culture. (g–i) Neurite cable length, number of branches, and average branch length over time. Data plotted as mean ± SD where *P < 0.05, **P < 0.01, ***P < 0.001, and ****P < 0.0001. Adapted and reprinted with permission from Ref [82].

As an alternative, dynamic covalent chemistry covers a range of subsets of reactions that enable reversible covalent crosslinking between biopolymers, which include Schiff base reactions [95, 96], borate bonds, reversible Diels–Alder reactions [97], and hydrazone bonds [98]. Heilshorn et al. [98] mixed hydrazine‐modified elastin‐like protein (ELP) and aldehyde‐ or benzaldehyde‐modified HA to enhance hydrogel stability through dynamic covalent crosslinking of hydrazone bonds. The kinetics of on/off formation of different speeds by different reactive groups ultimately confer hydrogels with different stress relaxation rates. This system of hydrogels was finally used for research on the reactions of NPCs to different viscoelastic microenvironments, and it was found that fast relaxation in the hydrogel promotes NPC maturation ([82]; Figure 4). Liu et al. [99] modified HA with aldehyde group and applied imine bonding to crosslink it dynamically with collagen, and hydrogels with different viscoelasticity were obtained by adjusting the concentration of aldehyde group, and interestingly, the change in aldehyde group concentration did not affect the stiffness of the hydrogels, and the stiffness was following the mechanical properties of brain tissue. Implanting this system of viscoelastic hydrogel into the site of TBI injury not only induces stem cells to migrate and recruit to the site of injury but also promotes the differentiation of stem cells into neurons to achieve the purpose of neural repair after brain injury [99]. Therefore, viscoelastic hydrogels have a broad application prospect in TBI nerve repair.

**FIGURE 5:**
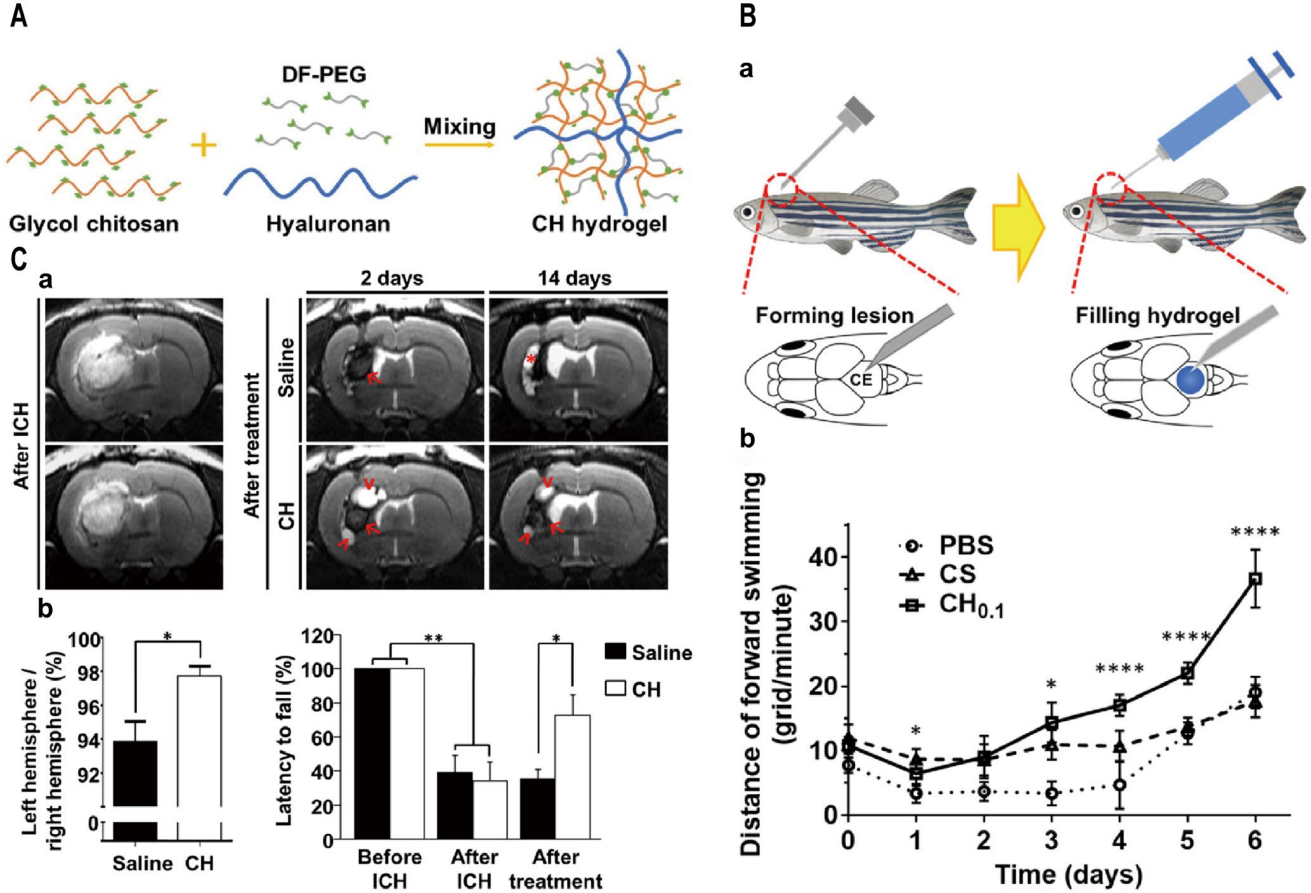
Preparation of dynamically crosslinked hydrogels based on Schiff base reaction for TBI treatment with promoted neurological functional recovery. (A) Schematic representation of the preparation of chitosan‐sodium alginate (CH) hydrogel prepared by mixing ethylene glycol chitosan solution with difunctional polyethylene glycol (DF‐PEG) and HA. (B) CH hydrogel implantation in a TBI zebrafish model promotes motor function recovery in adult zebrafish after implantation. (C) CH hydrogel alleviates brain atrophy and neurological deficits in a rat model of cerebral hemorrhage (ICH). Two‐way ANOVA was applied for comparison. *,***, and **** each represent *p* < 0.05, *p* < 0.001, and *p* < 0.0001. Adapted and reprinted with permission from Ref [101].

**FIGURE 6:**
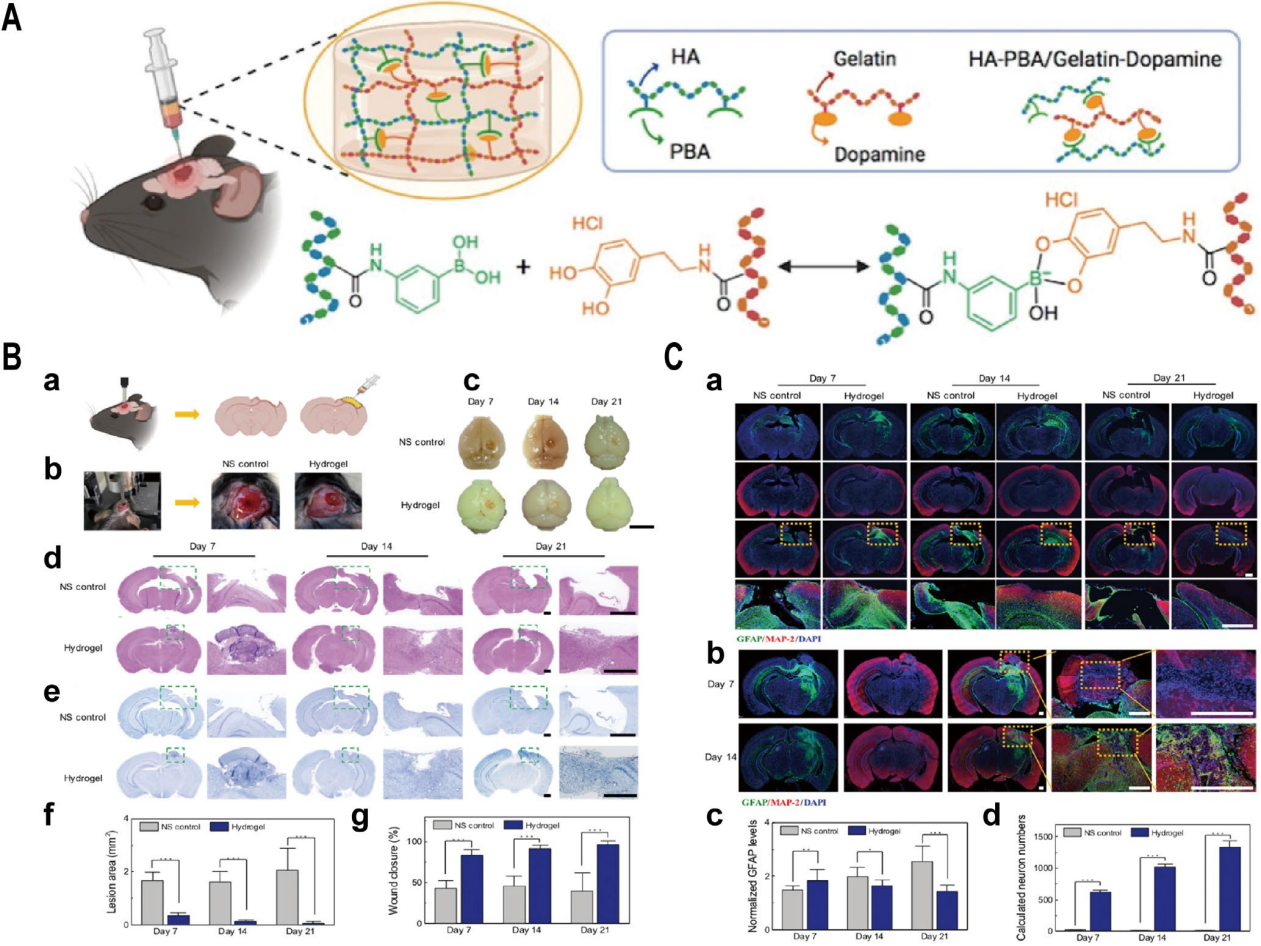
Hydrogel matched brain tissue stiffness and viscoelasticity to treat TBI, reduce scarring, and promote nerve repair. (A) Schematic diagram of hydrogel preparation. Preparation of viscoelastic hydrogels via borate bonds formed by the reaction of phenylboronic acid groups with catechol groups. (B) Histological analysis of regenerative brain tissue. (d, e) Hematoxylin–eosin (H&E), Nissl staining, and (f, g) quantification of the lesion area demonstrated that viscoelastic hydrogels have a facilitating effect on wound healing. (c) Scale bar: 5 mm; (d, e) Scale bar: 2 mm. (C) Increased astrocytes around the lesion cavity after hydrogel injection and migration of nerve cells into the lesion cavity. (a, b) Immunofluorescence staining for related proteins, (c) Quantitative analysis of GFAP, and (d) analysis of the number of neurons in the lesion cavity. (a) Scale bars, 1 mm. (b) Scale bars, 500 μm. Data plotted as mean ± SD where * *p* < 0.05, ** *p* < 0.01, and *** *p* < 0.001 by two‐tailed Student’s t‐test. Adapted and reprinted with permission from Ref [103].

Kalow et al. [100] created a couple of branching polymers and utilized AZO photosensitive switches for extrinsic control of the reactivity of dynamic covalent crosslinkers to reversibly stiffen/soften hydrogels. Besides, the preparation of DNH based on the dynamic crosslinking of the Schiff base reaction was evaluated in a TBI zebrafish model and a cerebral hemorrhage rat model, which provided a permissive microenvironment for axon growth and had a therapeutic effect on the cerebral injury cavity ([101]; Figure 5). Moreover, Zhang and teammates developed an injectable antioxidant hydrogel based on the Schiff base reaction of oxidized dextran (Odex) and gallic acid–conjugated gelatin (GGA), for the treatment of TBI [102]. The hydrogel is mixed with nerve cells and brain‐derived neurotrophic factors and sent to the site of brain injury to improve nerve regeneration and promote behavioral recovery [53, 54, 55]. In addition, the application of dynamic borate bonding to prepare DNH with viscoelasticity for injection into brain injury sites reduces scar formation and promotes neural repair ([103]; Figure 6). Although plenty of chemical strategies including noncovalent and dynamic covalent chemistry mentioned above prepare DNH, their applications in brain tissues or TBI treatments are still relatively rare, which may need to be more widely tailored with ability of sustained drug or cytokines delivery for further promoting TBI therapy.

## 4 Conclusion and Future Prospectives

TBI, as one of the highly prevalent diseases around the world, leads to various changes local physical microenvironment around the lesion after injury. For now, the clinical treatment of severe TBI is still not effective and has a poor prognosis. As a result, hydrogels have received great attention in terms of their application as delivery platforms for drugs or cells and as soft materials to fill cavities for the treatment of TBI. We illustrate the mechanical cues and their alteration during TBI, and summarize the current chemical strategies of dynamic hydrogels including DSH and DNH, to construct an in vitro model of TBI to match the brain tissue stiffness change during TBI, as well as the DNH to mimic the viscoelasticity of the brain for TBI treatments, respectively. The dynamic hydrogel preparation strategy will dynamically adjust the mechanics and internal network of the hydrogel to better match the dynamic mechanical changes in in vivo tissues. Dynamic network changes in the treatment of TBI enable nerve cells to better perceive spatial network changes in the biological niche and promote neural repair. In conclusion, compared with the previous single static hydrogels, dynamic hydrogels have a more novel and promising application prospect in TBI‐related simulation and clinical treatment.

However, there are many challenges in translating dynamic hydrogels into clinical applications. On the one hand, hydrogels must be biocompatible in clinical applications to avoid triggering immune reactions or inflammation. Natural biomaterials, such as collagen, gelatin, and silk protein, are excellent choices for preparing hydrogels with good biocompatibility. However, to further enhance the safety of these hydrogels, advancements in synthesis techniques and precise control of conditions are necessary. For instance, in the case of pH‐responsive and thermosensitive hydrogels, the selection of appropriate pH levels and temperatures is crucial, as the survival of tissues and cells in vivo depends on a precise physiological environment.

Besides, hydrogels prepared by using UV light have potential toxicity to cells. Although near‐infrared (NIR) light has been introduced in some studies to mitigate this toxicity, the penetration ability of both UV and NIR light is limited. Furthermore, precise control over the type, concentration, and position of the photosensitive moiety is essential to ensure the effectiveness and safety of these hydrogels. To address these challenges and improve the versatility of light‐responsive dynamic hydrogels, multiwavelength orthogonal photochemistry can be employed. This technique allows for the introduction of multiple wavelength‐responsive moieties, enabling independent regulation of the on/off state of the light. This, in turn, allows for more precise and high‐end dynamic properties of the hydrogels. Such hydrogels can even be used to study the effects of different properties on cells and tissues simultaneously.

Another critical aspect to consider is the stability and degradation behavior of dynamic hydrogels in vivo, which can be affected by noncovalent interactions. Therefore, in‐depth studies on the long‐term effects and toxicity of these hydrogels in vivo are necessary. Finally, the host reaction triggered by the implantation of hydrogels into living tissues poses a significant challenge in the clinical application of dynamic hydrogels. This reaction can lead to inflammation, rejection, or other adverse effects. Therefore, understanding and mitigating this host reaction are crucial for the successful clinical application of these hydrogels.

Implantable hydrogel‐induced host reactions are one of the main potential side effects to be considered in the clinical application of dynamic hydrogels. Chronic inflammation and fibrosis are major contributors to implant failure, and addressing these issues is crucial for the successful application of hydrogels in medical treatments [104]. To suppress the inflammatory reaction after hydrogel implantation, several strategies can be employed: (1) The ECM plays a crucial role in regulating cellular activity during immune response and tissue repair. By designing hydrogels that mimic the ECM, it is possible to minimize the host response by either enhancing or inhibiting normal immune cell function. This approach can help to create a more biocompatible environment for the hydrogel, reducing the likelihood of adverse reactions [105]. (2) Another effective strategy is to use hydrogels to co‐load anti‐inflammatory substances with neural stem cells and transport them to the site of brain injury. This approach has been shown to reduce neuroinflammation after TBI. By delivering both therapeutic agents locally through the hydrogel, it is possible to achieve a more targeted and effective reduction in inflammation [106]. (3) The degradability of the hydrogel is also an important factor in minimizing the host response. Hydrogels that degrade too quickly may be unstable and unable to achieve the desired modeling and therapeutic effects. Conversely, hydrogels that do not degrade can induce tissue scarring and glial cell formation, which can impede the nerve repair process [107]. Therefore, selecting a hydrogel with appropriate degradability is crucial for achieving successful treatment outcomes. In addition to these strategies, other approaches such as optimizing the hydrogel's surface properties, incorporating immunomodulatory molecules, and using cell‐derived matrices may also be effective in reducing the host response to hydrogel implantation. Overall, addressing the host reaction is a complex and multifaceted challenge that requires a combination of strategies to achieve successful clinical outcomes.

Dynamic hydrogels have a unique viscoelastic and dynamic network structure that gives them flexibility and reparability. However, in clinical applications, there is a need to ensure that hydrogels maintain stable mechanical properties under complex in vivo environments (e.g., body fluids and mechanical loading) and can support tissue repair or drug delivery durably and effectively. On the other hand, the mechanical properties and degradation rate of dynamic hydrogels can be controlled by precisely modulating the noncovalent bonds in a laboratory setting. However, it is a great challenge to maintain this tunability in the in vivo environment, especially to achieve personalized regulation in different patients, lesion sites, and therapeutic needs.

The preparation of dynamic hydrogels that can simultaneously simulate the stiffness and viscoelasticity changes in TBI is a technically challenging yet important area of research. The brain tissue is viscoelastic, and the simulation of its post‐TBI viscoelasticity with dynamic hydrogels is currently understudied. This is due to the complex interplay between mechanical cues and the crosslinking exchange rate, which makes it difficult to independently control their reaction kinetics dynamically. To address this challenge, one potential approach is to combine noncovalent bonding with dynamic covalent bonding to prepare hydrogels with dynamic stiffness and viscoelasticity that conform to the mechanical properties of brain tissues. This could involve the use of ECM‐derived biomaterials, such as collagen, HA, fibrin, and gelatin, which are known for their biocompatibility and ability to mimic the natural environment of cells. Brain acellular ECM is even more appealing; a lot of studies have identified that dynamic hydrogel prepared by applying brain dECM retains tissue‐specific components, including key structural and functional proteins, and that these unique biochemical features can stimulate brain repair by promoting angiogenic responses [108], neurological spectrum differentiation potentials [109], improving the formation of neural network structures [110], and increasing the capacity for cell adhesion and proliferation [111]. However, decellularization‐based therapies are less commonly used in TBI than in spinal cord injuries, and therefore, dynamic hydrogels for brain acellular ECM bases need to be explored more.

By incorporating both types of bonding into the hydrogel structure, it may be possible to achieve a more comprehensive simulation of the pathological changes in TBI. For example, noncovalent bonding could be used to provide the initial stiffness and stability of the hydrogel, while dynamic covalent bonding could be used to adjust the viscoelastic properties in response to changes in the microenvironment. However, it is important to note that the preparation of such hydrogels requires careful consideration of the material properties, reaction conditions, and crosslinking mechanisms to ensure that the resulting hydrogel is both safe and effective for use in TBI modeling and treatment.

While dynamic hydrogels hold vast promise for the modeling and treatment of TBI, another major obstacle to be addressed in translating this range of hydrogels from in vitro experiments to clinical applications is regulatory issues. Hydrogels are considered Class III medical devices by new European regulations. Biomaterials should ensure that human life and health are safeguarded, making the safety and efficacy of dynamic hydrogels the main indicators of regulation. The State Drug Administration has initiated part of the program containing new biomaterials for medical device applications, while the main focus is on applied research in osteoinduction and bone regeneration [112]. Considering the complexity and importance of the brain, dynamic hydrogels for the treatment of brain injuries need to be more uniform and strictly regulated. Due to the complex crosslinking mechanisms and mechanical properties of dynamic hydrogels, it is difficult to define and test the criteria for these hydrogels. This means that a large number of clinical trials and long‐term follow‐up studies are needed. Small‐scale synthesis of dynamic hydrogels in the laboratory is relatively easy to control, but to achieve clinical applications, large‐scale production processes must be developed to ensure product consistency and stability [113].

In conclusion, the preparation of dynamic hydrogels that simulate the stiffness and viscoelasticity changes in TBI is a complex and challenging task. However, by combining noncovalent bonding with dynamic covalent bonding and using ECM‐derived biomaterials, it may be possible to achieve a more comprehensive simulation of the pathological changes in TBI and contribute to the development of more effective treatment strategies.

## Conflicts of Interest

The authors declare no conflicts of interest.

## Data Availability

Data sharing is not applicable to this article as no new data were created or analyzed in this study.
